# Protective effect of *Lactobacillus salivarius* Li01 on thioacetamide‐induced acute liver injury and hyperammonaemia

**DOI:** 10.1111/1751-7915.13629

**Published:** 2020-07-11

**Authors:** Liya Yang, Xiaoyuan Bian, Wenrui Wu, Longxian Lv, Yating Li, Jianzhong Ye, Xianwan Jiang, Qing Wang, Ding Shi, Daiqiong Fang, Jingjing Wu, Kaicen Wang, Qiangqiang Wang, Jiafeng Xia, Jiaojiao Xie, Yanmeng Lu, Lanjuan Li

**Affiliations:** ^1^ State Key Laboratory for Diagnosis and Treatment of Infectious Diseases National Clinical Research Center for Infectious Diseases Collaborative Innovation Center for Diagnosis and Treatment of Infectious Diseases The First Affiliated Hospital College of Medicine Zhejiang University Hangzhou 310003 China; ^2^ The First Affiliated Hospital Wenzhou Medical University Wenzhou China

## Abstract

The gut microbiota plays pivotal roles in liver disease onset and progression. The protective effects of *Lactobacillus salivarius* Li01 on liver diseases have been reported. In this study, we aimed to detect the protective effect of *L. salivarius* Li01 on thioacetamide (TAA)‐induced acute liver injury and hyperammonaemia. C57BL/6 mice were separated into three groups and given a gavage of *L. salivarius* Li01 or phosphate‐buffered saline for 7 days. Acute liver injury and hyperammonaemia were induced with an intraperitoneal TAA injection. *L. salivarius* Li01 decreased mortality and serum transaminase levels and improved histological liver damage caused by TAA. Serum inflammatory cytokine and chemokine and lipopolysaccharide‐binding protein (LBP) concentrations, nuclear factor κB (NFκB) pathway activation and macrophage and neutrophil infiltration into the liver were significantly alleviated by *L. salivarius* Li01. *L. salivarius* Li01 also reinforced gut barrier and reshaped the perturbed gut microbiota by upregulating Bacteroidetes and *Akkermansia* richness and downregulating Proteobacteria, *Ruminococcaceae_UCG_014* and *Helicobacter* richness. Plasma and faecal ammonia levels declined noticeably in the Li01 group, accompanied by improvements in cognitive function, neuro‐inflammation and relative brain‐derived neurotrophic factor (BDNF) gene expression. Our results indicated that *L. salivarius* Li01 could be considered a potential probiotic in acute liver injury and hepatic encephalopathy (HE).

## Graphical abstract

*Lactobacillus salivarius* Li01 improved TAA‐induced acute liver injury and hyperammonemia which was mediated by restoring destroyed gut microbiota and barrier function, improving TAA‐induced liver damage and systemic inflammation, and alleviating hyperammonaemia, neuro‐inflammation and damaged cognitive function.

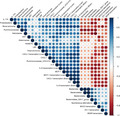

## Introduction

Acute liver failure (ALF) is characterized by explosive hepatocyte injury that can be caused by numerous insults, such as drug toxicity, hepatitis viral infection and autoimmune hepatitis (Bernal *et al*., 2015). ALF and its complications frequently produce fatal outcomes in previously healthy people. Although many advanced therapeutic strategies for the treatment of patients with ALF have been proposed, liver transplantation is always the most effective method but is used in only approximately 30% of ALF patients (Stravitz and Lee, 2019) and is limited by a shortage in liver donors (O'Grady, 2012). ALF still presents a high mortality rate (Wlodzimirow *et al*., 2012) and seriously threatens human health.

Hepatic encephalopathy (HE) is a major complication of ALF and an important predictor of prognosis. After recovery from ALF, neuropsychological dysfunction might persist (Stravitz and Lee, 2019), which can affect the quality of life. The precise pathogenesis of HE is still unclear. Hyperammonaemia has generally been considered the main driver of HE (Gorg *et al*., 2013). Furthermore, the concentration of plasma ammonia is associated with the severity of HE (Patidar and Bajaj, 2015) and outcome of ALF (Yanny *et al*., 2019).

Accumulating evidence has demonstrated that an intimate relationship exists between the intestinal microbiota and liver disease (Wiest *et al*., 2017). Normally, the intestinal microbiota can protect the host against potential pathogens and their components, such as lipopolysaccharide (LPS), through a mechanism termed colonization resistance (Litvak and Baumler, 2019). Lipopolysaccharide‐binding protein (LBP) is a stable index that reflects LPS exposure (Asada *et al*., 2019). When the liver experiences injury, the balance of the gut microbiota is destroyed, which may participate in the onset and progression of liver injury. Deaminase and urease activation in the intestinal microbiota represent the major external sources of ammonia (Nicaise *et al*., 2008). The non‐absorbable disaccharide lactulose and poorly absorbed antibiotic rifaximin are the most common treatments for HE and function by decreasing ammonia production and absorption separately. However, the therapeutic effects are not obvious, and side‐effects, such as bloating, nausea and infection, cannot be ignored (Gluud *et al*., 2016; Yanny *et al*., 2019).

Previous studies have demonstrated that lactobacilli can efficiently treat hyperammonaemia and HE by remodelling the intestinal microbiota and reducing the production and absorption of ammonia (Nicaise *et al*., 2008; Singh *et al*., 2018). *Lactobacillus salivarius* Li01 (CGMCC 7045) found in healthy humans can significantly alleviate carbon tetrachloride (CCL4)‐induced liver cirrhosis and d‐galactosamine‐induced liver injury (Lv *et al*., 2014; Shi *et al*., 2017). In addition, *L. salivarius* Li01 exhibits tolerance to bile stress and possesses antibacterial and antifungal effects (Lv *et al*., 2017).

Thioacetamide (TAA), a classic hepatotoxic agent, has been extensively used for several years to develop liver injury models by bioactivating inducible nitric oxide synthase (INOS) and nuclear factor κB (NFκB), which can lead to inflammatory cytokine expression and centrilobular necrosis (Amanzada *et al*., 2014). Moreover, after TAA injection, the resulting neurobehavioral abnormalities resemble HE in patients (Shen *et al*., 2015).

*Lactobacillus salivarius* Li01 has been confirmed to improve liver cirrhosis and liver injury, and HE is a major complication of liver cirrhosis and liver injury (Lv *et al*., 2014; Shi *et al*., 2017). However, the protective effects of *L. salivarius* Li01 on hyperammonaemia are unclear. Therefore, we conducted this study to detect the therapeutic value of *L. salivarius* Li01 in TAA‐induced acute liver injury and hyperammonaemia and the potential mechanisms.

## Results

### L. salivarius Li01 reduced mortality and liver damage in a TAA‐induced mouse model

**Fig. 1:**
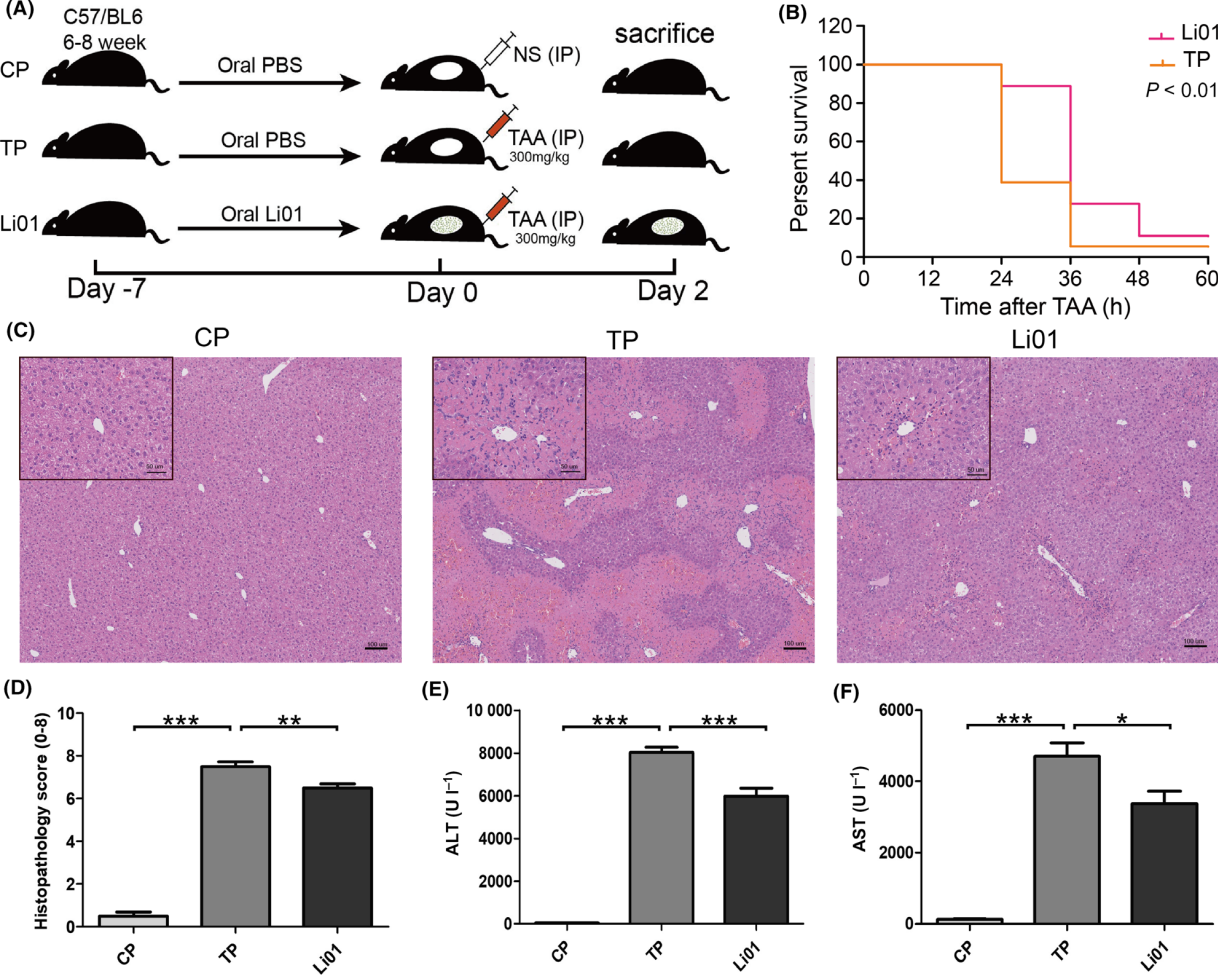
*Lactobacillus salivarius* Li01 intervention prolonged survival and improved liver injury. (A) Experimental scheme. CP group, normal mice treated with PBS; TP group, liver‐damaged mice treated with PBS; Li01 group, liver‐damaged mice treated with *L. salivarius* Li01. (B) Kaplan–Meier survival curves for the TP and Li01 groups, which were given high‐dose TAA. (C) Representative H&E staining of the liver. (D) Pathological scores of livers. (E) Serum levels of ALT. (F) Serum levels of AST. Data are presented as the mean ± SEM. *P < 0.05, **P < 0.01, ***P < 0.001.

We administered a high‐dose TAA treatment to mice pretreated with phosphate‐buffered saline (PBS) or *L. salivarius* Li01 to detect the influence of *L. salivarius* Li01 on the mortality of TAA‐induced acute liver injury. The results indicated that the mice pretreated with *L. salivarius* Li01 had lower mortality than the mice pretreated with PBS (log‐rank test *P* < 0.01; Fig. 1B). After the administration of a lower dose of TAA, four of the ten mice in the positive control group (TP group, treated with TAA + PBS) died. However, the mortality in the probiotic intervention group (Li01 group, treated with TAA + *L. salivarius* Li01) was 20%, which was lower than the 40% mortality rate observed in the TP group. Haematoxylin and eosin (H&E) staining of liver sections showed evident destruction of the liver architecture, inflammatory cell infiltration and necrosis in the periportal areas, while *L. salivarius* Li01 could rehabilitate this type of liver damage (Fig. 1C). Consistent with the H&E staining, the dramatically elevated serum alanine aminotransferase (ALT; *P* < 0.001) and aspartate aminotransferase (AST; *P* < 0.001) levels induced by TAA could also be significantly reduced by *L. salivarius* Li01 (*P* < 0.001 and *P* < 0.05 respectively; Fig. 1E and F). The above results indicated that *L. salivarius* Li01 could improve TAA‐induced liver damage.

### L. salivarius Li01 pretreatment changed the structure of the gut microbiota

To explore the influence of *L. salivarius* Li01 on the structure of the gut microbiota, we conducted 16S rRNA gene sequencing of mouse faeces collected before the TAA injection. The rarefaction curve shown in Fig. S1 indicated that the amount of sequencing data is reasonable.

**Fig. 2:**
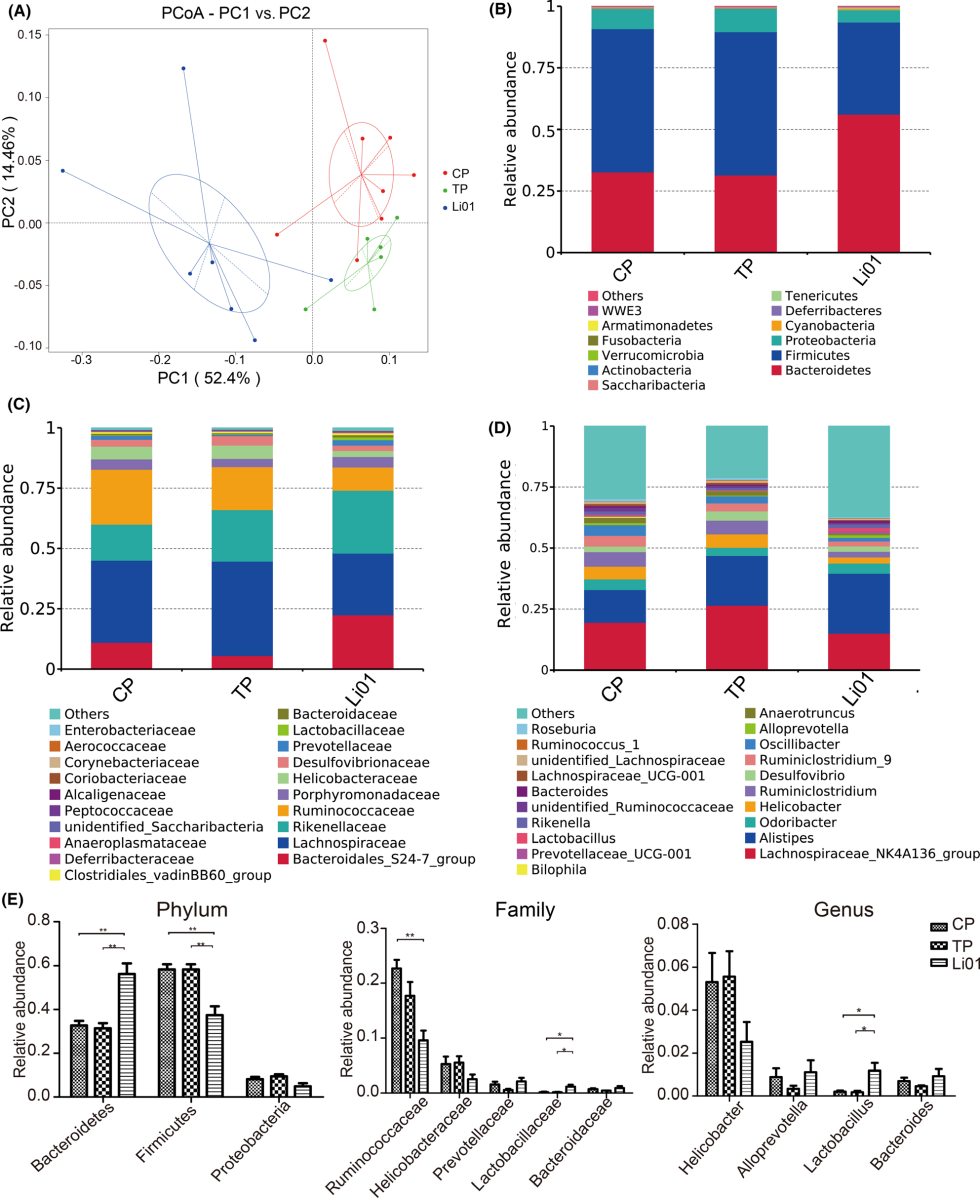
*Lactobacillus salivarius* Li01 intervention changed the structure of gut microbiota. (A) PCoA plot based on the weighted UniFrac metric. (B) Relative abundance of abundant taxa at phylum level. (C) Relative abundance of top twenty most abundant taxa at family level. (D) Relative abundance of top twenty most abundant taxa at genus level. (E) The specific taxa abundance at phylum (left), family (middle), genus (right) levels. Data are presented as the mean ± SEM. *P < 0.05, **P < 0.01, ***P < 0.001.

After the *L. salivarius* Li01 intervention, the α‐diversity of the gut commensals did not significantly change compared with that in the TP group (data not shown). The microbiome structure variance among the three groups was tested by UniFrac PCoA, permutational multivariate analysis (PERMANOVA or Adonis) and multiple response permutation procedure (MRPP). The *L. salivarius* Li01 intervention significantly changed the gut microbiome structure (Fig. 2A, Tables S2 and S3). The PCoA image showed that the microbiome clusters in the Li01 group were clearly distinct from those in the CP (treated with vehicle + PBS) and TP groups.

We detected the taxonomic abundance at the phylum, family and genus levels among the three groups (Fig. 2B–E). According to the results, compared with either the CP group or TP group, the *L. salivarius* Li01 supplementation significantly upregulated the relative abundance of Bacteroidetes and downregulated the richness of Firmicutes. Although there was no significant difference of Proteobacteria richness after the *L. salivarius* Li01 treatment, an elevated trend was observed. After the *L. salivarius* Li01 pretreatment, Ruminococcaceae, Helicobacteraceae and *Helicobacter* exhibited downward trends, and the richness of Lactobacillaceae and *Lactobacillus* was significantly increased.

### L. salivarius Li01 pretreatment reshaped the aberrations in the gut microbiota caused by the TAA administration

To explore the change in the intestinal microbiota in the TAA‐induced mouse model of acute liver injury, we conducted 16S rRNA gene sequencing of mouse faeces collected 48 h after the TAA administration. The rarefaction curve reflected that the sequencing depth was reasonable (Fig. S2).

**Fig. 3:**
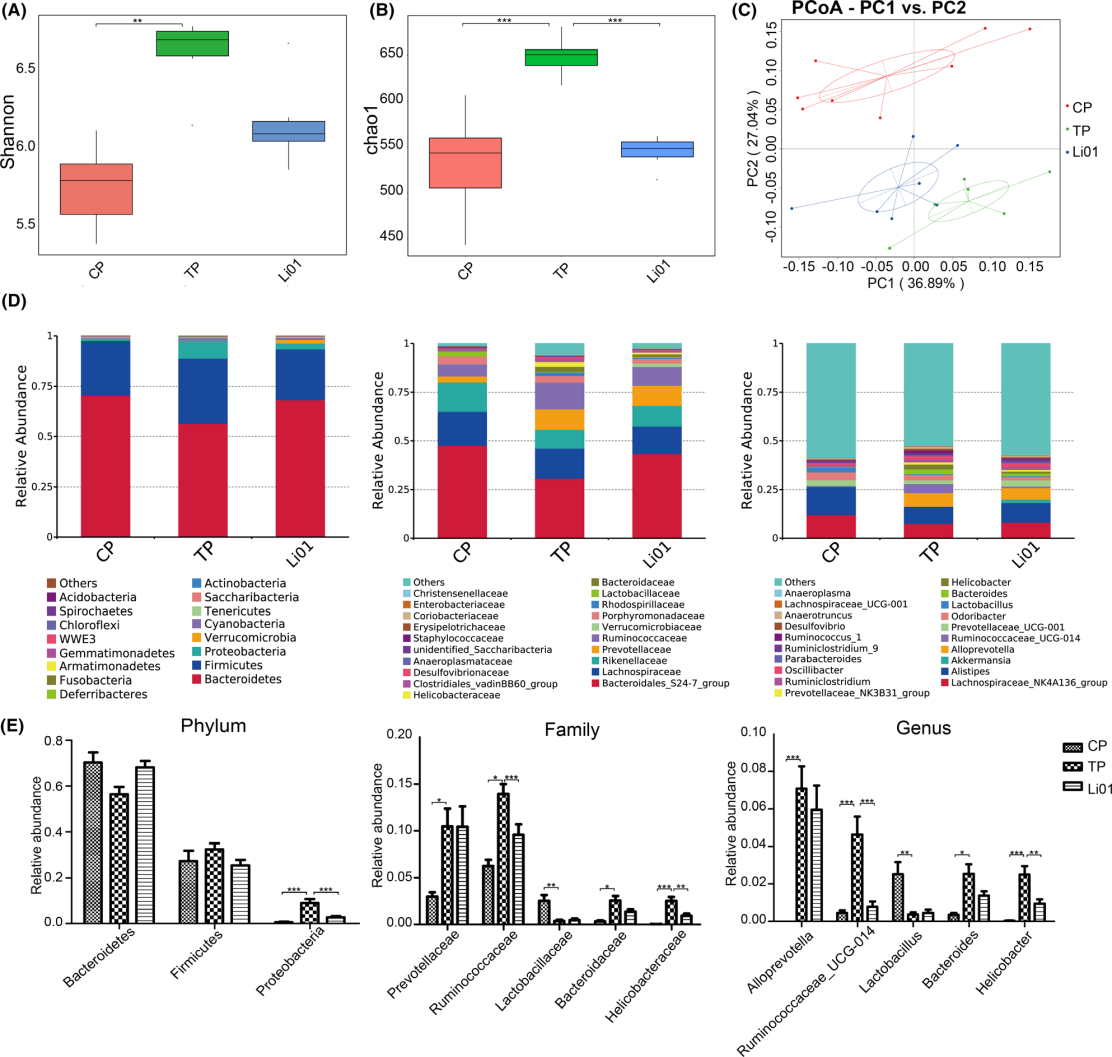
Alterations in the gut microbiota after TAA injection. (A) Shannon index. (B) Chao1 metric. (C) PCoA plot based on the weighted UniFrac metric. (D) Relative abundance of top twenty most abundant taxa at phylum (left), family (middle), genus (right) levels and bar charts of the specific taxa abundance. Data are presented as the mean ± SEM. *P < 0.05, **P < 0.01, ***P < 0.001.

The α‐diversity of the gut microbiota in each of the three groups after the TAA administration was calculated by the Shannon index and Chao 1 metric. The TAA treatment increased the α‐diversity of gut commensals as indicated by noticeably increased Shannon and Chao 1 indices (*P* < 0.001 Fig. 3A and B). Compared with the TP group, the Li01 group exhibited reduced α‐diversity according to the Chao 1 indices.

There were marked differences in the commensal composition among the three groups after TAA injection (Tables S4 and S5). The PCoA image shows that the microbiome clusters of the three groups were clearly separated (Fig. 3C). The microbiome profile of the CP group was noticeably different from those of the other two groups. However, the distance in the microbiome profile between the Li01 group and CP group was shorter than that between the TP group and CP group, which corresponded with the R2 value and expected delta. The results indicated that the TAA injection could change the structure of the intestinal microbiota and that *L. salivarius* Li01 could partially restore the disordered microbiota.

At the phylum level, the TAA injection obviously increased the relative abundance of Proteobacteria (Fig. 3D, *P* < 0.001). However, compared with the TP group, the richness of Proteobacteria in the Li01 group was significantly decreased. At the family level, there were significant higher the relative abundances of Prevotellaceae, Ruminococcaceae, Bacteroidaceae, and Helicobacteraceae in the TP group than those in the CP group after the TAA injection (*P* < 0.05, *P* < 0.05, *P* < 0.05 and *P* < 0.001 respectively). The *L. salivarius* Li01 treatment dramatically reduced the relative abundances of Ruminococcaceae and Helicobacteraceae (*P* < 0.001 and *P* < 0.01). At the genus level, the TAA injection significantly increased the relative abundances of *Alloprevotella*, *Ruminococcaceae_UCG‐014*, *Bacteroides* and *Helicobacter* (*P* < 0.001, *P* < 0.001, *P* < 0.05 and *P* < 0.001 respectively), but compared with the TP group, the relative abundances of *Ruminococcaceae_UCG‐014* and *Helicobacter* in the Li01 group were significantly reduced (*P* < 0.001 and *P* < 0.01). In addition, the TAA injection could significantly reduce the relative abundances of Lactobacillaceae and *Lactobacillus* (*P* < 0.01).

**Fig. 4:**
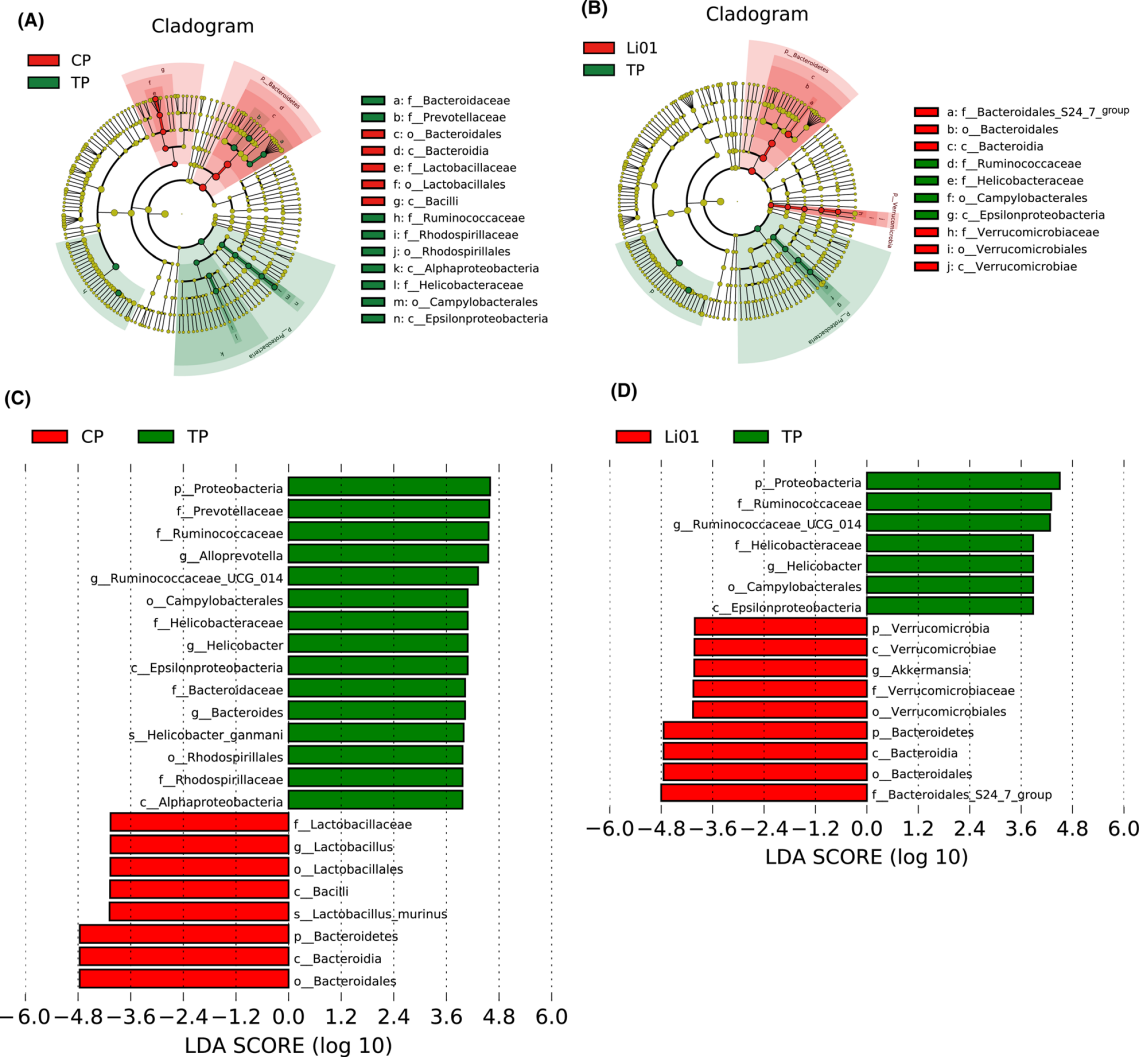
Effects of pretreatment with *Lactobacillus salivarius* Li01 on the alterations of gut bacterial taxonomic abundance after TAA injection. (A and B) LEfSe cladogram. (C and D) Discriminative biomarkers with an LDA score > 3.8. Red represents the CP or Li01 group, and green represents the TP group.

To further explore the key bacteria associated with TAA‐induced acute liver injury and the *L. salivarius* Li01 treatment, we conducted an LEfSe analysis (Fig. 4A‐D). The mice in the CP and TP groups had higher relative abundances of Bacteroidetes and Proteobacteria respectively. The families Ruminococcaceae (containing the genus *Ruminococcaceae_UCG_014*) and Prevotellaceae and the genus *Alloprevotella* were enriched in the TP group compared with those in the CP group. The other taxa enriched in the TP group, such as the order Campylobacterales, family Helicobacteraceae and genus *Helicobacter*, all belonged to the phylum Proteobacteria. In addition, the TAA injection could significantly reduce the abundance of the genus *Lactobacillus*. However, after the *L. salivarius* Li01 intervention, the reduced levels of the phylum Bacteroidetes and upregulated enrichment of the phylum Proteobacteria and family Ruminococcaceae were partially restored. In addition, *Akkermansia* plays a protective role in many liver diseases, such as alcoholic liver disease, non‐alcoholic fatty liver disease and immune‐mediated liver injury (Wu *et al*., 2017; Zhu *et al*., 2017; Grander *et al*., 2018). The *L. salivarius* Li01 administration increased the abundance of the genus *Akkermansia*. The results indicated that the *L. salivarius* Li01 intervention could reshape the disorder in the microbiota induced by TAA.

### L. salivarius Li01 intervention partially restored the gut barrier function

**Fig. 5:**
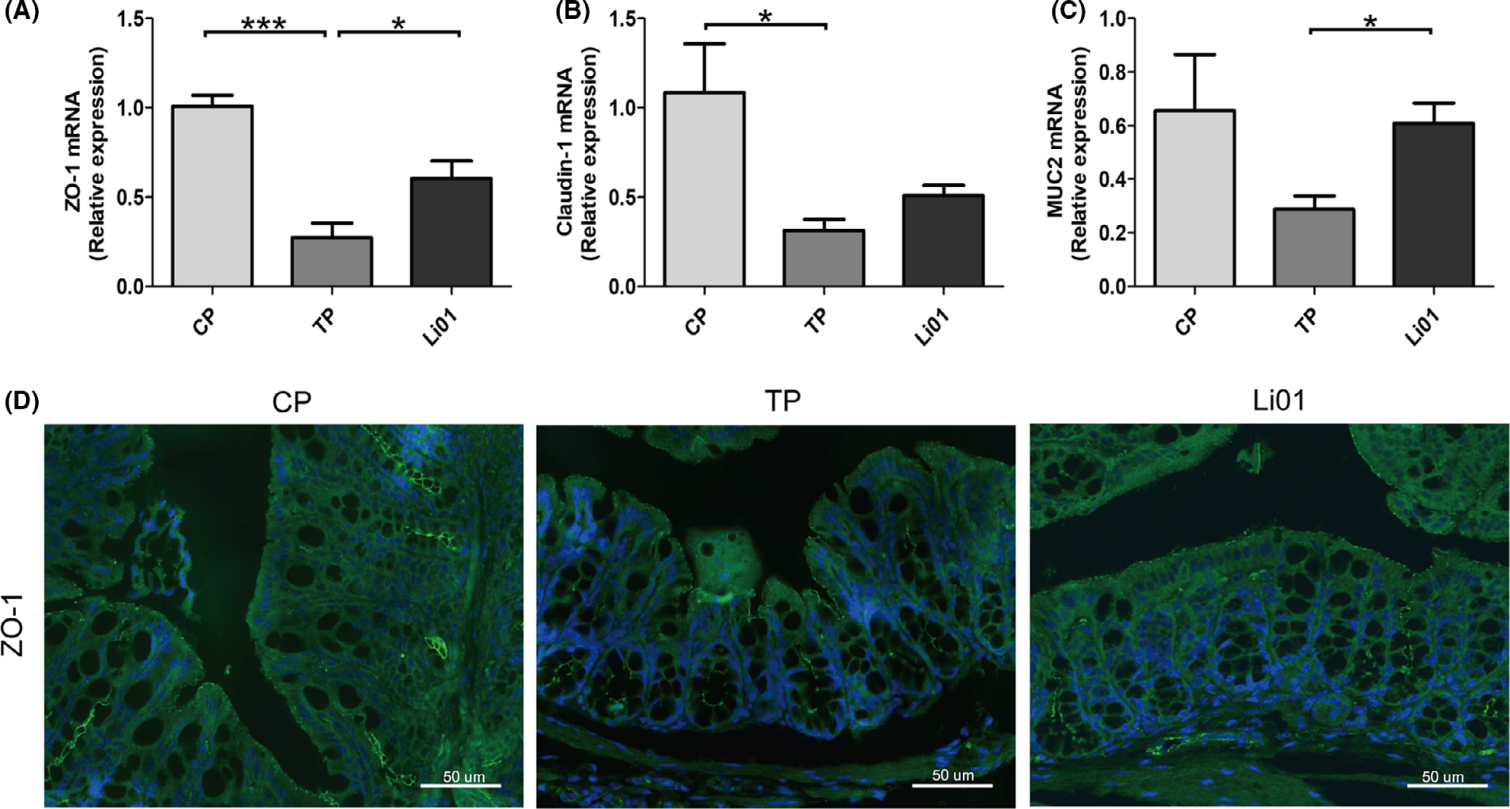
*Lactobacillus salivarius* Li01 reinforced gut barrier function. (A–C) Relative mRNA expression of ZO‐1, Claudin‐1 and MUC2 in the colon. (D) Representative immunofluorescence staining for ZO‐1 in the colon (scale bar: 50 µm). Data are presented as the mean ± SEM. *P < 0.05, **P < 0.01, ***P < 0.001.

The overgrowth of gut bacteria and damage to the intestinal barrier has been shown to be correlated with liver injury caused by TAA (Harputluoglu *et al*., 2012). Hence, we detected the colonic mRNA expression of zonula occludens‐1 (ZO‐1), claudin‐1 and mucin2 (MUC2) to assess the gut barrier function. The TAA administration destroyed the gut barrier as evidenced by a decreased expression of ZO‐1 and claudin‐1 (*P* < 0.001 and *P* < 0.05 respectively; Fig. 5A and B); however, the *L. salivarius* Li01 intervention ameliorated this impact as indicated by significant improvements in the ZO‐1 and MUC2 transcriptional levels (*P* < 0.05). Although the upregulation of claudin‐1 expression was not significant, an increasing trend existed. To further test our hypothesis, we conducted immunofluorescence staining for ZO‐1(Fig. 5D). This result was consistent with the mRNA expression results for ZO‐1 in the colon, indicating that the breakdown of ZO‐1 induced by TAA was partially restored by the *L. salivarius* Li01 intervention.

### L. salivarius Li01 attenuated the serum LBP levels and the TAA‐induced activation of the NFκB pathway

**Fig. 6:**
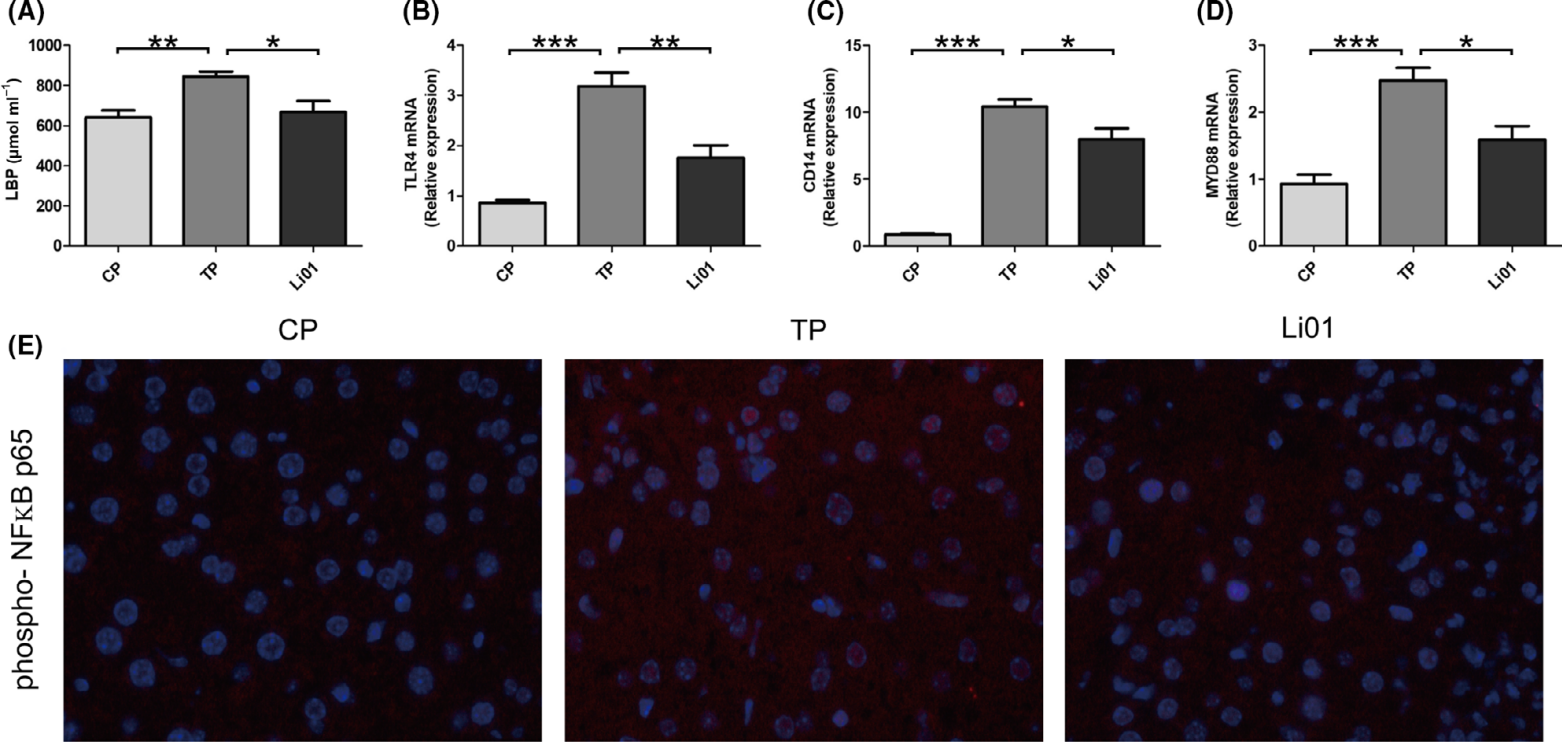
*Lactobacillus salivarius* Li01 alleviated serum LBP levels and suppressed the activation of the NFKB pathway. (A) Serum LBP levels. (B) Relative hepatic mRNA expression of TLR4, CD14 and MYD88. (C) Representative immunofluorescence staining for phospho‐NFκB p65 in the liver (scale bar: 50 µm). Data are presented as the mean ± SEM. *P < 0.05, **P < 0.01, ***P < 0.001.

When the gut barrier is damaged, microorganisms and their components, represented by LPS, break through the gut barrier. LPS is related to the onset and progression of liver diseases and can activate the NFκB pathway via Toll‐like receptor 4 (TLR4) and myeloid differentiation 88 (MyD88). LBP is a stable index that reflects LPS exposure (Asada *et al*., 2019). Therefore, we detected the serum concentration of LBP. The results showed that the LBP levels in the Li01 group were significantly lower (Fig. 6A, *P* < 0.05) than those in the TP group, indicating that *L. salivarius* Li01 could improve the upregulation of the LPS level induced by TAA (*P* < 0.01, Fig. 4A). In addition, the relative mRNA expression of TLR4, CD14 (a co‐receptor of TLR4) and MyD88 was assessed by RT‐PCR and could be downregulated by the administration of *L. salivarius* Li01 (*P* < 0.01, *P* < 0.05 and *P* < 0.05 respectively; Fig. 6B–D). In addition, we assessed phospho‐NFκB p65 nuclear translocation in the liver by immunofluorescence. We found that after the TAA administration, mice had an increased fluorescence intensity for phospho‐NFκB p65 in the nucleus (Fig. 4E). Moreover, *L. salivarius* Li01 could downregulate the nuclear translocation of phospho‐NFκB p65 induced by the TAA injection. Therefore, we hypothesized that *L. salivarius* Li01 could improve the LPS levels and downregulate NFκB pathway activation to alleviate TAA‐induced acute liver injury.

### L. salivarius Li01 mitigated the TAA‐induced inflammatory response and recruitment of macrophages and neutrophils into the liver

**Fig. 7:**
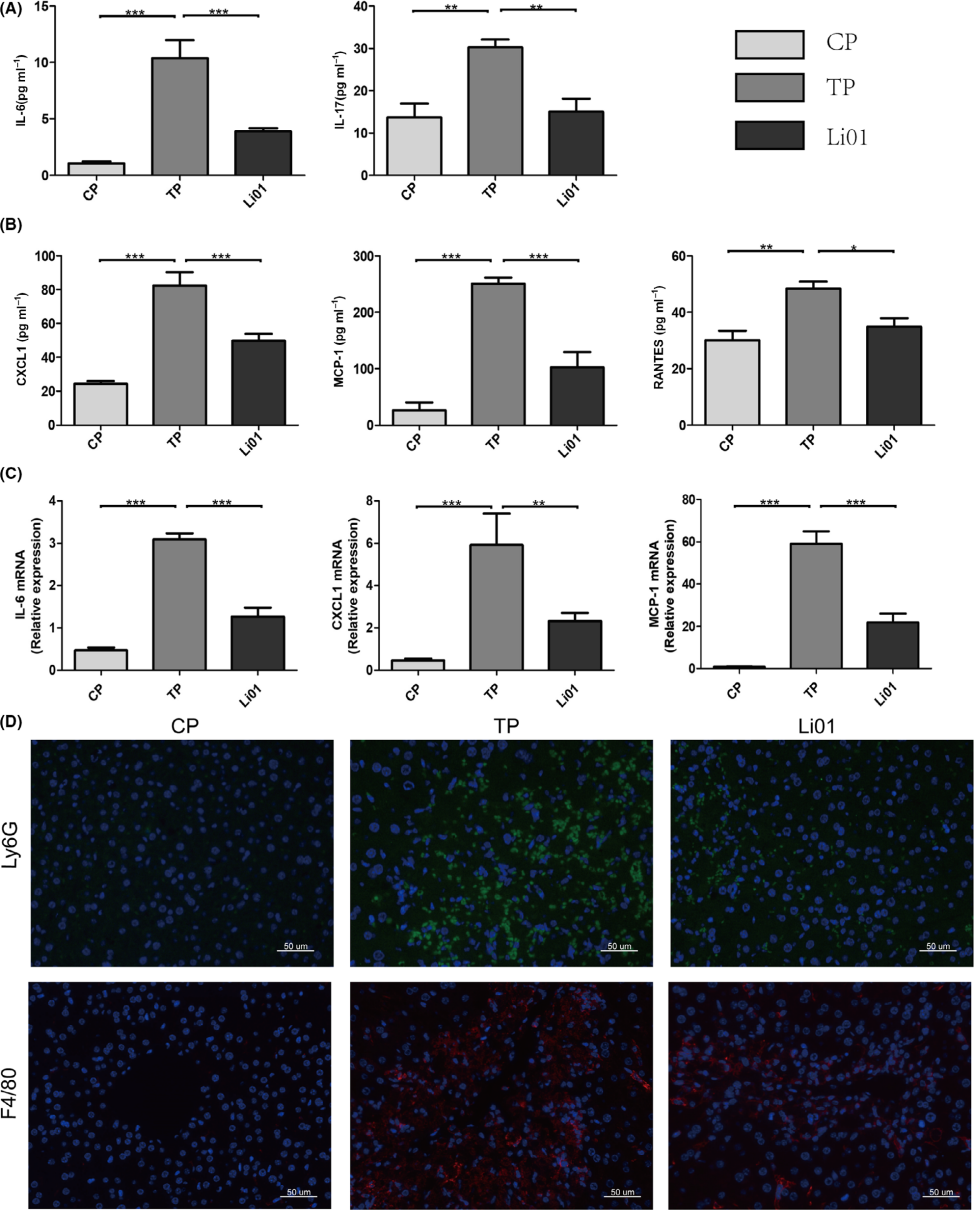
*Lactobacillus salivarius* Li01 relieved TAA‐induced systemic and hepatic inflammation, and reduced the hepatic recruitment of macrophages and neutrophils. (A) Expression of the cytokines IL‐6 and IL‐17A in the serum. (B) Expression of the chemokines CXCL1, MCP1 and RANTES in the serum. (C) Relative mRNA expression of IL‐6, CXCL1 and MCP1 in the liver. (D) Representative immunofluorescence staining for F4/80 (macrophage marker) and Ly6G (neutrophil marker) in the liver. Scale bar: 50 µm. Data are presented as the mean ± SEM. *P < 0.05, **P < 0.01, ***P < 0.001.

Thioacetamide induces the activation of the NFκB pathway, leading to the expression of pro‐inflammatory factors and chemokines (Shapiro *et al*., 2006). Can *L. salivarius* Li01 improve TAA‐induced inflammation? To address this question, we investigated the pro‐inflammatory cytokine and chemokine mRNA levels in the liver. The mice in the Li01 group exhibited a significantly lower relative expression of Interleukin‐6 (IL‐6), monocyte chemotactic protein 1(MCP1) and chemokine (C‐X‐C motif) ligand 1(CXCL1) than the mice in the TP group (*P* < 0.001, *P* < 0.001 and *P* < 0.01 respectively; Fig. 7C). In addition, we detected the serum levels of some pro‐inflammatory cytokines. Forty‐eight hours after the TAA challenge, the serum concentrations of pro‐inflammatory factors (Interleukin (IL)‐17A and IL‐6) and chemokines (MCP‐1, CXCL1 and RANTES) were significantly upregulated (Fig. 7A and B). However, compared with those in the TP group, the cytokine concentrations in the Li01 group were evidently mitigated.

Thioacetamide injection can induce the accumulation of macrophages and neutrophils in the liver (Amanzada *et al*., 2014). MCP1 and RANTES can regulate macrophage recruitment, and CXCL1 is a chemoattractant of neutrophils. In our study, *L. salivarius* Li01 improved the upregulation of the levels of these chemokines. Whether *L. salivarius* Li01 can decrease the infiltration of macrophages and neutrophils requires further exploration. For this reason, we conducted immunofluorescence staining for macrophages (F4/80+) and neutrophils (Ly6G+) in the liver, and the images show that the TAA injection could promote macrophage (F4/80+) and neutrophil (Ly6G+) migration into the liver (Fig. 7D). Moreover, in the Li01 group, the infiltration of macrophages and neutrophils was sporadic.

### L. salivarius Li01 intervention led to reductions in blood and faecal ammonia concentrations and neuro‐inflammation levels and restored cognitive function

**Fig. 8:**
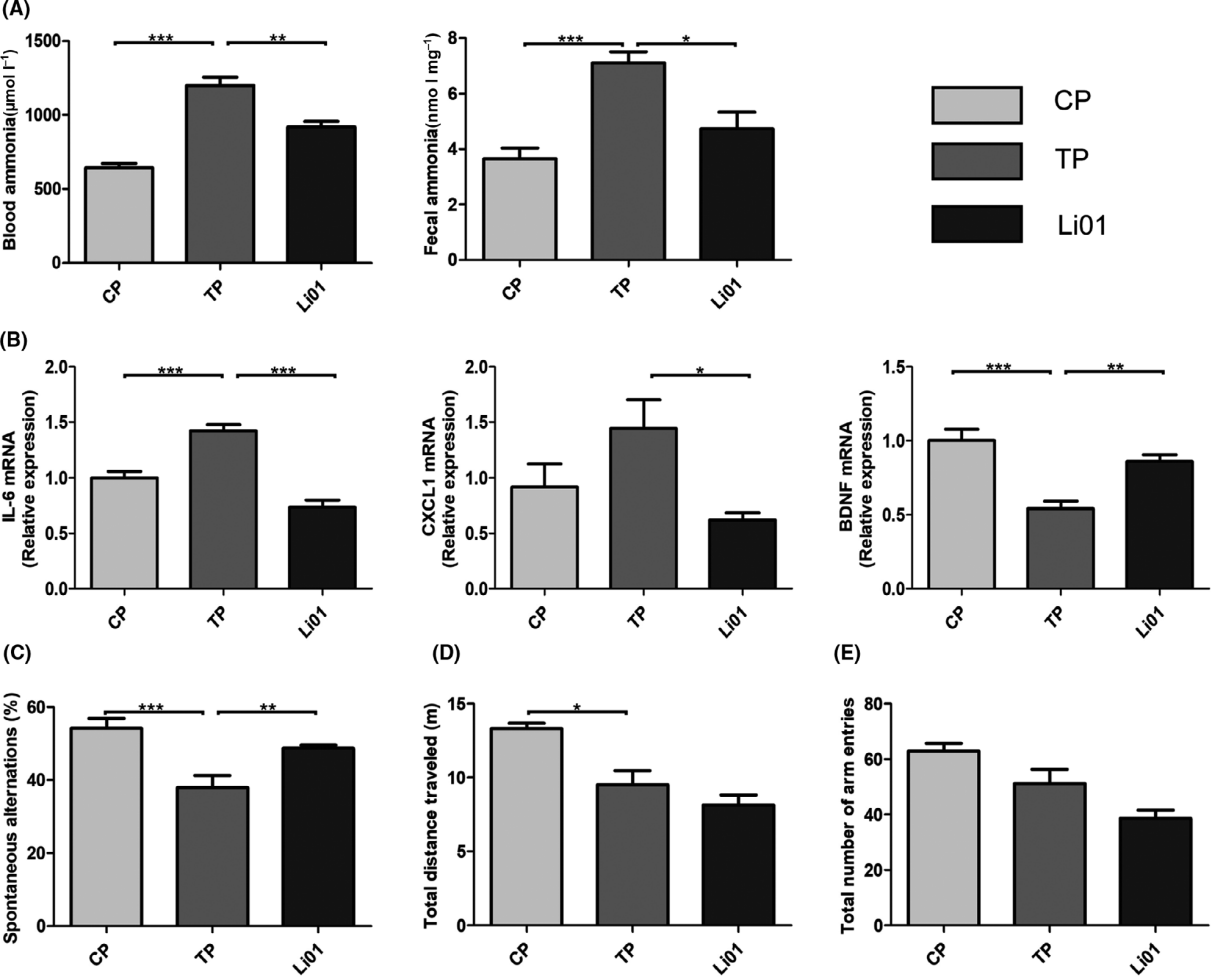
*Lactobacillus salivarius* Li01 downregulated plasma and faecal ammonia levels and improved neuro‐inflammation and cognitive function. (A) Plasma and faecal ammonia levels. (B) Relative expression of the IL‐6, CXCL1 and BDNF genes in the cortex. (C) Testing of spontaneous alternation with a Y maze at 48 h after TAA administration. (D) Total distance travelled. (E) Total number of arm entries within 8 min. Data are presented as the mean ± SEM. *P < 0.05, **P < 0.01, ***P < 0.001.

Acute liver injury is often accompanied by hyperammonaemia, which is closely related to HE (Blei *et al*., 2001; Nicaise *et al*., 2008). The gut is regarded as an important source of circulating ammonia (van de Poll *et al*., 2008). Therefore, the faecal and plasma ammonia concentrations were tested. The blood and faecal ammonia levels were significantly higher in the TP group than those in the CP group (*P* < 0.001 and *P* < 0.001 respectively; Fig. 8A). However, after the *L. salivarius* Li01 administration, the faecal ammonia levels were evidently decreased (*P* < 0.05). Consistent with the improvement in the faecal ammonia level, the reduction in the blood ammonia level was also significant (*P* < 0.01).

The relative mRNA expression of IL‐6 and CXCL1 in the cortex increased after the TAA administration (Fig. 8B), whereas the *L. salivarius* Li01 intervention drastically improved this impact (*P* < 0.001 and *P* < 0.05 respectively).

Brain‐derived neurotrophic factor (BDNF) plays a pivotal role in not only the growth and survival of neurons (Barde *et al*., 1982; Leibrock *et al*., 1989) but also the acquisition, maintenance and recall of spatial memory (Mizuno *et al*., 2003). As shown in Fig. 8B, the TAA administration noticeably downregulated BDNF mRNA expression in the cortex (*P* < 0.001); however, the downregulation of BDNF expression was attenuated by the *L. salivarius* Li01 intervention (*P* < 0.001).

We conducted a Y maze test to evaluate the cognitive impairment caused by TAA and the protective effect of *L. salivarius* Li01. Our results illustrate that compared with the CP group, the TAA challenge significantly decreased spontaneous alternations in the TP group (*P* < 0.001, Fig. 8C). However, *L. salivarius* Li01 had a significant protective effect on these spontaneous alternations (*P* < 0.05). The locomotor activity of the mice was quantified by measuring the total arm entries and distance travelled. The mice in the CP group travelled a markedly longer distance than those in the TP group (*P* < 0.05). Nevertheless, the mice treated with either *L. salivarius* Li01 or PBS did not display a sufficient difference in the total distance travelled. In addition, the total arm entries did not change after the TAA administration or *L. salivarius* Li01 treatment. *L. salivarius* Li01 possessed protective effects on only cognitive function in the mice but had no beneficial effect on locomotor activity.

### Correlations between the gut microbiota and gut barrier markers and systemic, hepatic and neuro‐inflammation

**Fig. 9:**
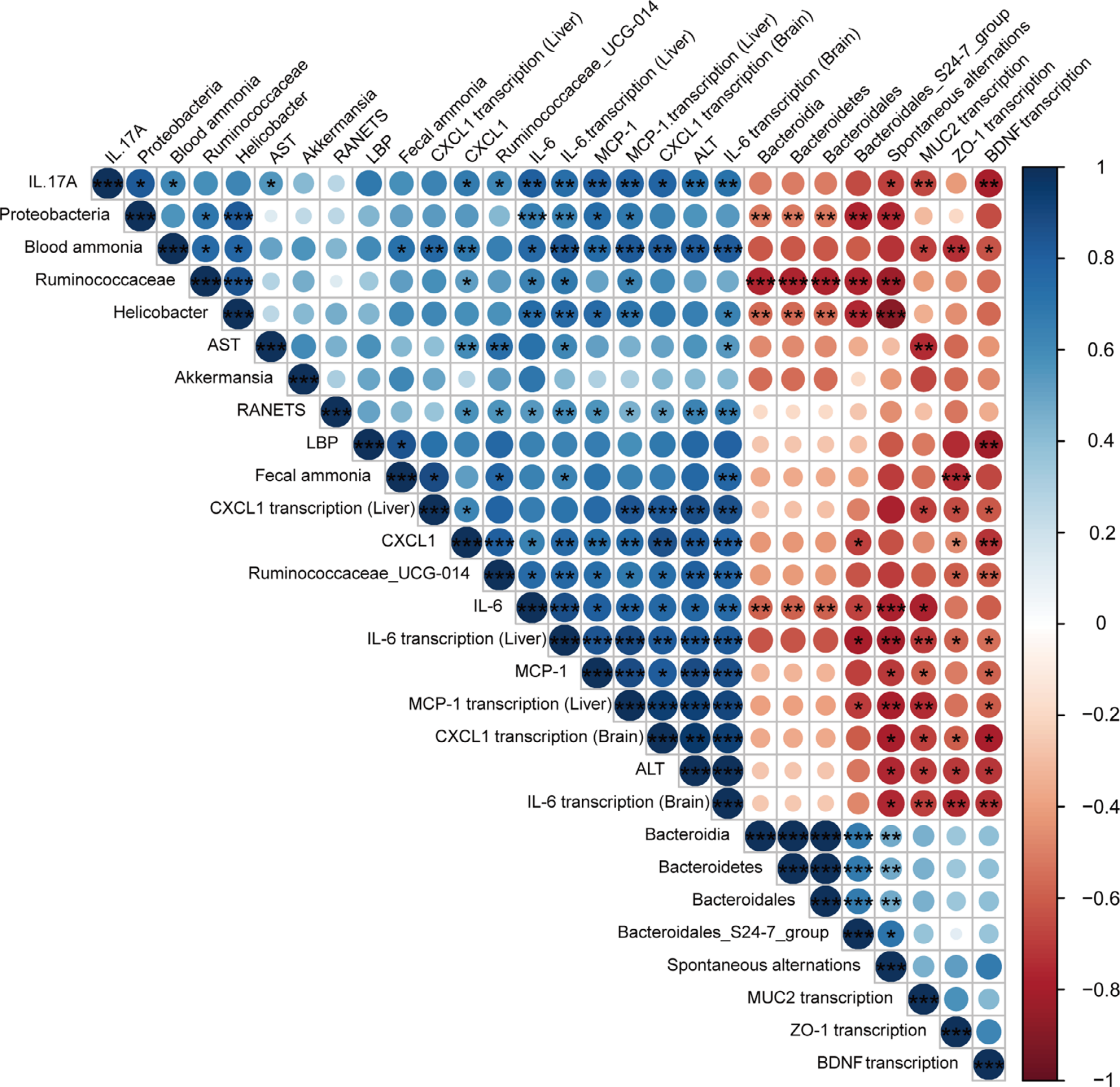
Correlation analysis. Spearman rank correlation analysis was used to detect associations between the discriminative gut microbiota and injury‐related parameters. The colour key and circle size show the strength of the correlation. Dark blue represents a positive correlation; red represents a negative correlation. *P < 0.05, **P < 0.01, ***P < 0.001.

To further assess the influence of the altered gut microbiota, we conducted a correlation analysis between discriminative bacterial genera and injury parameters (Fig. 9). Markers of systemic and hepatic inflammation, including IL‐6 and MCP‐1, were significantly positively correlated with the relative abundances of Proteobacteria, *Helicobacter* and *Ruminococcaceae_UCG.014* (*P* < 0.05), which were enriched in the TP group. In addition, the population density of the Bacteroidales_S24.7_group, which was prominently abundant in the Li01 group, was negatively associated with the serum levels of CXCL1 and IL‐6 and the expression of the IL‐6 and MCP1 genes in the liver (*P* < 0.05). On the one hand, the plasma ammonia concentrations had a significant positive relationship with the ammonia levels in the faeces (*P* < 0.05) and negative relationships with the mRNA levels of MUC2 (*P* < 0.05) and ZO1 (*P* < 0.01) in the colon. On the other hand, the neuro‐inflammation parameters (IL‐6 and CXCL1 mRNA levels) and BDNF mRNA levels were closely tied to the blood ammonia levels, systemic inflammation, liver function and the enrichment in *Ruminococcaceae_UCG.014*. Additionally, the spontaneous alternations had negative correlations with Proteobacteria (*P* < 0.01); Ruminococcaceae (*P* < 0.01); *Helicobacter* (*P* < 0.001); serum levels of ALT, IL‐17A, IL‐6, MCP1 and CXCL1; and hepatic mRNA expression of IL‐6 and MCP1 and a positive correlation with the Bacteroidales_S24.7_group (*P* < 0.05). Therefore, we speculate that *L. salivarius* Li01 modified‐gut microbiota played a pivotal role in limiting the detrimental effects of the TAA challenge.

## Discussion

The perturbed gut‐liver‐brain axis has emerged as a pivotal component of liver diseases and complication HE (Wiest *et al*., 2017). Dysbiosis of the gut microbiota not only contributes to some bowel diseases but also influences the onset and progression of some extraintestinal diseases, including acute liver injury. Probiotic intervention is considered a potential therapeutic strategy.

Our results indicated that the *L. salivarius* Li01 pretreatment decreased mortality and alleviated liver injury by downregulating the serum transaminase levels and reducing the histological damage. The protective effects of *L. salivarius* Li01 partially depended on reshaping the intestinal microbiota, strengthening the intestinal barrier and improving systemic and hepatic inflammation. Moreover, *L. salivarius* Li01 ameliorated HE by alleviating liver damage and decreasing the plasma and faecal ammonia levels.

The gut microbiota, mucins, the continuously regulated turnover of intestinal epithelial cells and tight junction complexes are essential components of the gut barrier (Cliffe *et al*., 2005; McDole *et al*., 2012; Ramanan and Cadwell, 2016). Our results showed that the TAA administration destroyed the gut barrier as evidenced by the decreased expression of ZO‐1 and Claudin‐1, and the *L. salivarius* Li01 treatment restored the gut barrier manifested as the increase in ZO‐1 and MUC2. Microorganisms, microbial components and metabolites can all affect extraintestinal organs by disrupting the intestinal barrier (Nakamoto *et al*., 2017). *L. salivarius* Li01 reshaped the gut microbiota, including *Akkermansia* and *Alloprevotella*, which are associated with the gut barrier function (Bian *et al*., 2019; Xing *et al*., 2019). Our findings demonstrate that *L. salivarius* Li01 decreased the serum IL‐6, IL‐17, CXCL1, MCP1 and RANTES levels which may be a pivotal medium for damage to the gut barrier function (Soares *et al*., 2010). The effect of *L. salivarius* Li01 on reshaping the gut microbiota and alleviating systemic inflammation may contribute to restoring the destroyed gut barrier damaged by TAA.

Lipopolysaccharide, also known as endotoxin, is located in the bacterial cell wall and is a unique component of gram‐negative bacteria. Normally, LPS transfer to extraintestinal organs can be prevented by an intact intestinal barrier. However, in our TAA‐induced ALF mouse model, the gut barrier was damaged, and LPS could easily break through the impaired intestinal barrier as evidenced by the elevated serum LBP levels which is a stable index that reflects LPS exposure (Yang *et al*., 2017). LBP and CD14 are essential mediators of the recognition of LPS by the TLR4‐MD2 complex (Ryu *et al*., 2017). Therefore, the expression of TLR4 and CD14 in the liver was increased in TP group. Subsequently, the expression of MYD88 was increased in the TP group, MYD88‐dependent signalling was activated, the translocation of phosphorylated NFκB p65 into the nucleus was increased, and the NFκB signalling pathway was activated after the TAA treatment (Kagan and Medzhitov, 2006). However, *L. salivarius* Li01 restored the gut microbiota and barrier function, which may contribute to the lower level of serum LPS, and further activation of the NFκB signalling pathway induced by the TAA injection was inhibited as evidenced by the decreased serum LBP levels, hepatic TLR4, CD14 and MYD88 mRNA levels, and the translocation of phosphorylated NFκB p65 into the nucleus. The activation of the NFκB signalling pathway can induce the release of cytokines and chemokines and the recruitment of inflammatory cells (Amanzada *et al*., 2014). In our study, the relative mRNA abundances of IL‐6, MCP1 and CXCL1 in the liver and those of the systemic inflammatory parameters, including IL‐6, IL‐17, MCP1, KC and RANENTS, were upregulated after the TAA injection. Numerous chemokines and cytokines are secreted by Kupffer cells, liver myofibroblasts (LMFs) and biliary cells, which are mostly located in the hepatic portal area (Sprenger *et al*., 1997; Amanzada *et al*., 2014), which might explain why the liver damage mostly presented in the periportal tracts as shown by the H&E staining. Macrophages can both accentuate inflammation and mediate inflammation resolution and tissue repair during liver injury (Li *et al*., 2019). The generation of reactive oxygen species (ROS) is considered to contribute neutrophils to liver injury (Jaeschke and Hasegawa, 2006). Chemokines play an important role in recruiting inflammatory cells. MCP1, RANTES and KC are known chemoattractant of inflammatory cells. Our results showed that the infiltration of macrophages and neutrophils was increased, which was consistent with the elevations in the MCP1, RANTES and CXCL1 levels in the positive control group (TP group). However, after the *L. salivarius* Li01 intervention, the release of chemokines and recruitment of inflammatory cells were both significantly alleviated. Therefore, we speculated that the improvement in the gut barrier function by *L. salivarius* Li01 reduced the serum LPS level, inhibited the activation of the NFκB signalling pathway, and further alleviated systemic and hepatic inflammation, which may play an important role in the mechanism by which *L. salivarius* Li01 improved TAA‐induced acute liver injury.

The gut microbiota, which contains many organisms with different genomes, is considered a previously forgotten organ that plays important roles in physiological processes, such as nutrient digestion and self‐defence. In our study, we revealed that the TAA‐induced acute liver injury in our mouse model destroyed the intestinal microbiota homeostasis, which was accompanied by *Lactobacillus* deficiency. However, the *L. salivarius* Li01 intervention changed the structure of the gut microbiota and increased the relative abundance of *Lactobacillus*. The TAA injection favoured Proteobacteria and *Helicobacter*, which include many pathogens, have been related to liver injury in a previous study (Gu *et al*., 2020), and were positively correlated with systematic inflammation in our study. However, the *L. salivarius* Li01 pretreatment reduced these bacteria. A previous study demonstrated that *Alloprevotella* was associated with intestinal villus damage (Xing *et al*., 2019). Notably, the relative abundance of *Alloprevotella* was increased in the TP group in which the intestinal barrier function was damaged. In addition, *Ruminococcaceae_UCG‐014* was negatively correlated with the short‐chain fatty acid level in the faeces, which plays a protective role in non‐alcoholic steatohepatitis (Zhang *et al*., 2019). An overgrowth of *Ruminococcaceae_UCG‐014* was found in the TP group and was closely related to systemic inflammation and liver function. Supplementing with *L. salivarius* Li01 could recover the TAA‐induced depletion of Bacteroidetes and favour *Akkermansia*. Accumulating studies have elucidated that *Akkermansia*, a butyrate‐producing genus, has beneficial effects on liver damage and the gut barrier function (Wu *et al*., 2017; Bian *et al*., 2019). Our results demonstrate that *L. salivarius* Li01 reshaped the disturbed gut microbiota caused by the TAA treatment, which could alleviate acute liver injury.

Thioacetamide injection can induce acute progressive liver damage with parallel involvement of hyperammonaemia and is recognized as a reliable mouse model of acute liver injury and HE (Shen *et al*., 2015). However, the damaged liver cannot eliminate gut‐derived ammonia as evidenced by the positive relationship between the blood ammonia levels and the severity of HE in the clinic (Weissenborn *et al*., 2007). In our study, the decreased spontaneous alternations and expression of BDNF and the increased expression of IL‐6 in the TP group indicated that the TAA treatment‐induced neuro‐inflammation and destroyed cognitive function. Plasm ammonia was significantly upregulated in the TP group and closely correlated with neuro‐inflammation and the relative expression of the BNDF gene. Therefore, we speculate that hyperammonaemia plays an important role in HE. The gut microbiota can produce ammonia, which is regarded as a toxin from the gut associated with neurotoxicity based on highly convincing evidence (Norenberg, 1996). This theory was confirmed in our study by the elevated faecal ammonia level in the TP group and the positive correlation between blood and faecal ammonia. The common medicines used to treat hyperammonaemia include lactulose and rifaximin, and their mechanisms are based on regulating the intestinal microbiome (Gluud *et al*., 2016). However, the existing treatment schemes have some shortcomings that limit their clinical use. Interventions with probiotics are considered potentially effective as a treatment for HE. In this study, *L. salivarius* Li01 not only alleviated the TAA‐induced acute liver injury but also improved the complication HE as evidenced by the elevated spontaneous alternations and BDNF mRNA level and reduced expression of IL‐6 and CXCL1 in the brain. On the one hand, the correlation analysis showed the close correlation among blood ammonia, faecal ammonia and microbiota. The *L. salivarius* Li01 intervention restored the gut microbiota, which may contribute to the simultaneous declines in the blood and faecal ammonia levels. Accordingly, we hypothesized that *L. salivarius* Li01 decreases the level of gut‐derived ammonia to reduce the blood ammonia levels and further improve HE. On the other hand, as *L. salivarius* Li01 relieved the TAA‐induced liver injury, the ability to eliminate ammonia in the liver was improved.

Nevertheless, some limitations exist in our study. Although our results indicate that close relationships exist between the gut microbiota and some parameters of systemic, hepatic and neuro‐inflammation, the mechanisms need to be fully explored and verified.

## Conclusion

Our findings show the beneficial role of *L. salivarius* Li01 in TAA‐induced acute liver injury and HE in mice. We hypothesize that *L. salivarius* Li01 improves the damage induced by TAA via reshaping the gut microbiota, reinforcing the gut barrier function, downregulating NFκB pathway activation, alleviating systemic inflammation, reducing inflammatory cell infiltration into the liver and decreasing ammonia levels. Therefore, these results indicate that *L. salivarius* Li01 may be a potential probiotic that can be used against acute liver injury and its complication HE.

## Experimental procedures

### Strains and culture conditions

*Lactobacillus salivarius* Li01 isolated from the faeces of healthy individuals was grown anaerobically in MRS medium for 24 h at 37 °C (Lv *et al*., 2017). The cultures were centrifuged at 8000 *g* for 10 min. Then, the supernatant was discarded, and the precipitate was washed twice with sterile PBS and resuspended in PBS at a final concentration of 3 × 10^9^ CFUs ml^−1^ by testing the absorbance at 630 nm (O.D. range from 0.6 to 0.8; Bian *et al*., 2019). Fresh *L. salivarius* Li01 suspension was used in the animal experiment.

### Animals

Male C57BL/6 mice (6–8 weeks, Shanghai SLAC Laboratory Animal, Co, Ltd, Shanghai, China) were housed for 7 days for adaptation to conventional laboratory conditions (room temperature: 20–22 °C, 12‐h light/dark cycle, free access to food and drinking water). Subsequently, the mice were randomly assigned to three groups and treated with 0.2 ml vehicle (groups CP *n* = 8 and TP *n* = 10) or a fresh *L. salivarius* Li01 suspension (3 × 10^9^ CFUs ml^−1^, group Li01 *n* = 10) per day by intragastric infusion for one week (Fig. 1A). The acute liver injury and hyperammonaemia model in the TP and Li01 groups were induced via a single intraperitoneal (i.p.) injection of 300 mg kg^−1^ TAA (Sigma‐Aldrich, St. Louis, MO, United States). Before the TAA injection, a faecal sample was collected from each mouse. The Y maze neurobehavioral test was conducted 48 h after the TAA administration. Then, the mice were sacrificed, and the liver, colon, brain tissue, blood and intestinal contents were collected for the subsequent tests.

### Survival curves

The mice were given a single high dose of TAA (600 mg kg^−1^) by i.p. injection after 7 days of *L. salivarius* Li01 suspension (3 × 10^9^ CFUs ml^−1^ in PBS) or PBS administration. The survival time of the mice was recorded (Shen *et al*., 2015).

### Neurobehavioral test

The memory and spatial learning of the mice were assessed by the Y maze test, which was conducted with an apparatus with three identical black plastic arms. Normally, a mouse would investigate new arms. Before the test, the mice were acclimated to the testing environment for 30 min. Each mouse was released at the end of one arm and allowed to navigate freely for 8 min. All mice were placed in the same place. The total number of arm entries, distance travelled and spontaneous alternations (entering the three different arms sequentially) were recorded. The percentage of spontaneous alternations was defined by the following formula: [(number of alternations)/(total arm entries‐2)] × 100 (Shen *et al*., 2015; Ghafouri *et al*., 2016).

### Biochemical assays

The serum was separated from blood samples by centrifugation at 3000 *g* for 10 min at 4 °C. Alanine aminotransferase (ALT) and aspartate aminotransferase (AST) were evaluated with a dry chemistry analyser (FUJI DRI‐CHEM 7000V, FUJIFILM, Tokyo, Japan). The LBP levels were determined with LBP ELISA kits (Guduo, Shanghai, China). The serum cytokine levels were measured with magnetic bead suspension arrays (Bio‐Rad, Hercules, CA, USA).

### Plasma and faecal ammonia

Blood was treated with the anticoagulant heparin and centrifuged at 1000 *g* for 10 min at 4 °C to obtain plasma. The plasma ammonia concentrations were measured using an Ammonia Assay kit (Abcam, Cambridge, MA, USA). The faecal samples (100 mg) were suspended in 1 ml assay buffer, and the supernatants were obtained by centrifugation at 13 000 *g* for 10 min at room temperature (Shen *et al*., 2015). The faecal ammonia levels were detected according to the manufacturer's protocols.

### Histopathology and immunofluorescence

Appropriately sized samples were obtained from the left liver and colon and immediately fixed in 10% formalin for 24 h. Then, paraffin was used to embed the tissue. H&E staining and a histological activity index analysis were performed to estimate liver damage (Knodell *et al*., 1981).

The liver samples were cut into 4‐µm‐thick sections and stained with anti‐phospho‐p65, anti‐Ly6G, or anti‐F4/80 antibodies. The colon samples were cut and stained with anti‐ZO‐1 (Proteintech, Rosemont, IL, USA) antibodies according to a previously reported immunofluorescence protocol (Chung *et al*., 2014). The images were visualized by P250 FLASH (3D HISTECH, Budapest, Hungary).

### RNA extraction and real‐time PCR analysis

The liver cortex and colon samples were collected at sacrifice and stored at −80 °C. The total RNA was extracted by using a RNeasy Mini Kit (QIAGEN, Hilden, Germany). The detailed steps are described in the product manuals. PrimeScript RT Master Mix (TAKARA Biomedicals, Kusatsu, Japan) was applied to accomplish reverse transcription. The mRNA relative abundance was measured by SYBR Premix Ex Taq II reagent (TAKARA Biomedicals, Kusatsu, Japan) using an Applied Biosystems VIIA7 Real‐time PCR system (see Table S1 for the primer information). We included two biological replicates for each sample in the test. GAPDH was employed as an internal control for the liver and brain samples. β‐actin was used as the internal control for the colon samples.

### Analysis of the microbiota

Faecal pellets were collected before the TAA injection and sacrifice and immediately stored in a −80 °C freezer. We performed the stool DNA extraction with the QIAamp Fast DNA Stool Mini Kit (Qiagen, Hilden, Germany). Briefly, 300 µl 0.1 mm zirconia silica beads and 1 ml InhibitEX buffer were added to the samples, and then, the samples were homogenized twice at 5000 rpm for 30 s. The next steps referred to the manufacturer’s recommendations. The distinct region V3‐V4 of 16S rRNA was amplified using Phusion^®^ High‐Fidelity PCR Master Mix (New England Biolabs). The PCR products were mixed and then purified with a GeneJET™ Gel Extraction Kit (Thermo Scientific). Sequencing was performed using an Ion S5™ XL platform (Thermo Scientific). The data were split and filtered, and the chimaeras were removed to obtain clean reads. Clean reads with ≥ 97% sequence similarity were clustered into operational taxonomic units (OTUs) by Uparse software. The species annotation was performed using the SILVA database. The alpha diversity was analysed by QIIME (Version 1.7.0). The principal coordinate analysis (PCoA) was conducted with R software (Version 2.15.3) according to a distance matrix of weighted UniFrac. The linear discriminant analysis effect size (LefSe) analysis was performed online (Bajaj *et al*., 2014).

### Statistical analysis

The differences in the Kaplan–Meier survival curves were analysed by the log‐rank test. The data are expressed as the mean ± SEM. The significance of the differences in the ALT, AST, LBP, serum cytokine, plasma ammonia and faecal ammonia levels and relative abundances were determined by a one‐way analysis of variance (ANOVA). Tukey’s or Dunnett’s tests were conducted to adjust for multiple comparisons. PERMANOVA and MRPP were applied to analyse the β‐diversity of the gut microbiota. The correlations between different variables were analysed by a Spearman rank correlation analysis using R software. SPSS 20.0 (SPSS, Inc., Chicago, IL, USA) and GraphPad prism (GraphPad Software, Inc., CA, USA) were used to complete the statistical work. A *P*‐value < 0.05 was considered statistically significant.

## Conflict of interest

The authors declare that they have no competing interests.

## Supporting information


**Fig. S1.** The rarefaction curve reflected the sequencing depth of the faecal 16S rRNA sequencing analysis before TAA treatment.

**Fig. S2.** The rarefaction curve reflected the sequencing depth of the faecal 16S rRNA sequencing analysis after TAA treatment.

**Table S1.** Specific primers applied for RT‐PCR test.
**Table S2.** PERMANOVA test of community structure differences in the gut microbiota among groups after *L. salivarius* Li01 intervention.
**Table S3.** MRPP test of community structure differences in the gut microbiota among groups after *L. salivarius* Li01 intervention.
**Table S4.** PERMANOVA test of community structure differences in the gut microbiota among groups after TAA injection.
**Table S5.** MRPP test of community structure differences in the gut microbiota among groups after TAA injection.

## Data Availability

The 16S rRNA gene sequencing data were deposited in the sequence read archive (SRA) database (accession no. PRJNA636106).
