# Supplementary figures and images for: Protective effect of Lactobacillus salivarius Li01 on thioacetamide‐induced acute liver injury and hyperammonaemia
Source: Microb Biotechnol. 2020 Jul 11;13(6):1860–76. doi: 10.1111/1751-7915.13629 (PMC7533332; doi:10.1111/1751-7915.13629)

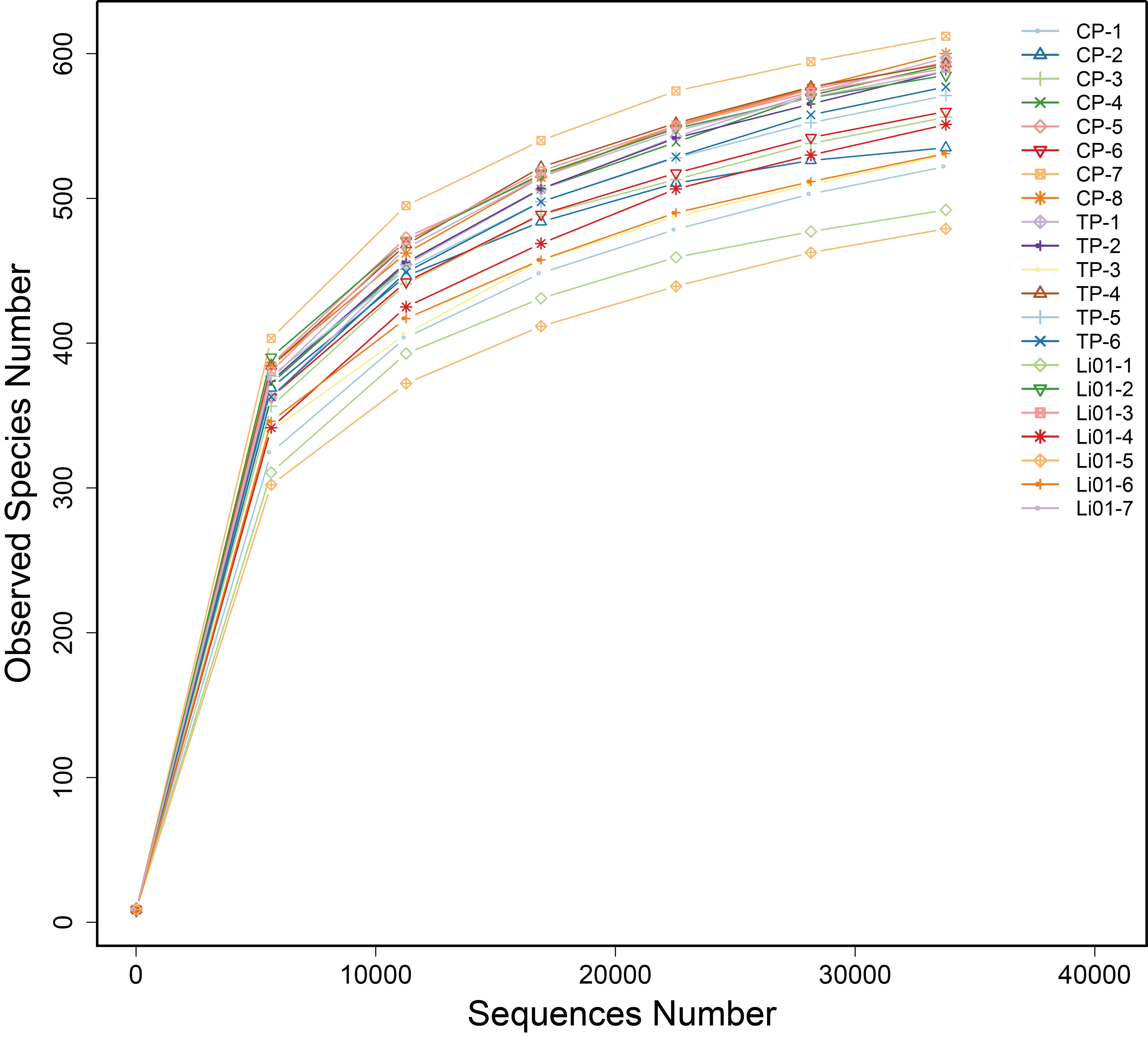

Supplement: Supplementary file 1 — Fig. S1. The rarefaction curve reflected the sequencing depth of the faecal 16S rRNA sequencing analysis before TAA treatment. [file MBT2-13-1860-s001.tif]

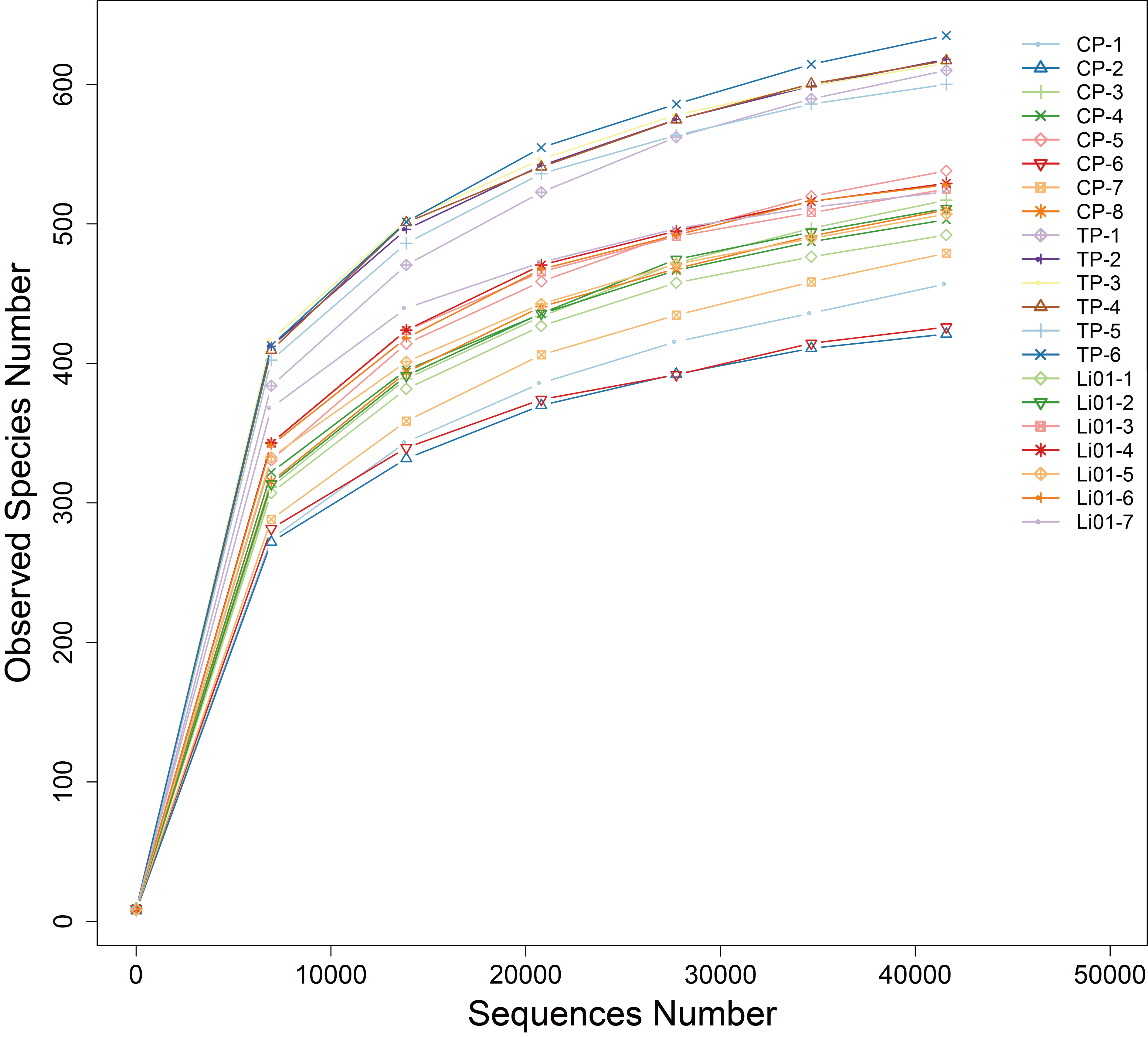

Supplement: Supplementary file 2 — Fig. S2. The rarefaction curve reflected the sequencing depth of the faecal 16S rRNA sequencing analysis after TAA treatment. [file MBT2-13-1860-s002.tif]
