# Supplementary material for: Protective effect of Lactobacillus salivarius Li01 on thioacetamide‐induced acute liver injury and hyperammonaemia
Source: Microb Biotechnol. 2020 Jul 11;13(6):1860–76. doi: 10.1111/1751-7915.13629 (PMC7533332; doi:10.1111/1751-7915.13629)
Supplement: Supplementary file 3 — Table S1. Specific primers applied for RT‐PCR test. Table S2. PERMANOVA test of community structure differences in the gut microbiota among groups after L. salivarius Li01 intervention. Table S3. MRPP test of community structure differences in the gut microbiota among groups after L. salivarius Li01 intervention. Table S4. PERMANOVA test of community structure differences in the gut microbiota among groups after TAA injection. Table S5. MRPP test of community structure differences in the gut microbiota among groups after TAA injection. [file MBT2-13-1860-s003.docx]

Supplementary Table S1. Specific primers applied for RT-PCR test

| Gene | Forward Sequence (5'-3') | Reverse Sequence (5'-3') |
| --- | --- | --- |
| β-actin | CATCCGTAAAGACCTCTATGCCAAC | ATGGAGCCACCGATCCACA |
| GAPDH | TGCGACTTCAACAGCAACTC | ATGTAGGCAATGAGGTCCAC |
| IL-6 | TAGTCCTTCCTACCCCAATTTCC | TTGGTCCTTAGCCACTCCTTC |
| MCP1 | TTAAAAACCTGGATCGGAACCAA | GCATTAGCTTCAGATTTACGGGT |
| CXCL1 | CTGGGATTCACCTCAAGAACATC | CAGGGTCAAGGCAAGCCTC |
| TLR4 | ATGGCATGGCTTACACCACC | GAGGCCAATTTTGTCTCCACA |
| CD14 | ACTTCTCAGATCCGAAGCCAG | CCGCCGTACAATTCCACAT |
| MYD88 | AGGACAAACGCCGGAACTTTT | GCCGATAGTCTGTCTGTTCTAGT |
| ZO-1 | GCCGCTAAGAGCACAGCAA | GCCCTCCTTTTAACACATCAGA |
| Claudin-1 | TGCCCCAGTGGAAGATTTACT | CTTTGCGAAACGCAGGACAT |
| MUC2 | ATGCCCACCTCCTCAAAGAC | GTAGTTTCCGTTGGAACAGTGAA |
| BNDF | TTACCTGGATGCCGCAAACAT | TGACCCACTCGCTAATACTGTC |

Supplementary Table S2. PERMANOVA test of community structure differences in the gut microbiota among groups after *L. salivarius* Li01 intervention.

| Group | Df | SumsOfSqs | MeanSqs | F.Model | R2 | Pr(>F) |
| --- | --- | --- | --- | --- | --- | --- |
| TP VS CP | 1(12) | 0.024721 (0.165132) | 0.024721 (0.013761) | 1.7964 | 0.13021 (0.86979) | 0.098 |
| TP VS Li01 | 1(11) | 0.1439 (0.1992) | 0.143904 (0.018109) | 7.9466 | 0.41942 (0.58058) | 0.002 |
| CP VS Li01 | 1(13) | 0.16191 (0.27195) | 0.16191 (0.02092) | 7.7397 | 0.37318 (0.62682) | 0.001 |

PERMANOVA: The permutational multivariate analysis of variance; Df: degree of freedom; SumsOfSqs: Total variance; MeanSqs: mean squares; R2: The ratio of the intragroup variance to the total variance. The larger the value of R2 is, the higher the degree of interpretation of the difference by group. Pr(>F): significance. The corresponding residual term values are shown in parentheses.

Supplementary Table S3. MRPP test of community structure differences in the gut microbiota among groups after *L. salivarius* Li01 intervention.

| Group | A | observed-delta | expected-delta | Significance |
| --- | --- | --- | --- | --- |
| TP VS CP | 0.02169 | 0.4729 | 0.4834 | 0.057 |
| TP VS Li01 | 0.053 | 0.4544 | 0.4798 | 0.013 |
| CP VS Li01 | 0.05123 | 0.5022 | 0.5293 | 0.001 |

MRPP: Multiple response permutation procedure; observed delta: intragroup difference; expected delta: intergroup difference. The larger the value of expected delta was, the greater the difference between the groups. A>0: intergroup difference>intragroup difference; A<0: intragroup difference>intergroup difference.

Supplementary Table S4. PERMANOVA test of community structure differences in the gut microbiota among groups after TAA injection.

| Group | Df | SumsOfSqs | MeanSqs | F.Model | R2 | Pr(>F) |
| --- | --- | --- | --- | --- | --- | --- |
| TP VS CP | 1(12) | 0.12965  (0.23914) | 0.129650  (0.019928) | 6.5059 | 0.35156  (0.64844) | 0.002 |
| TP VS Li01 | 1(11) | 0.044117  (0.182273) | 0.044117  (0.016570) | 2.6625 | 0.19487  (0.80513) | 0.019 |
| CP VS Li01 | 1(13) | 0.08425  (0.26953) | 0.084249  (0.020733) | 4.0635 | 0.23814  (0.76186) | 0.004 |

PERMANOVA: The permutational multivariate analysis of variance; Df: degree of freedom; SumsOfSqs: Total variance; MeanSqs: mean squares; R2: The ratio of the intragroup variance to the total variance. The larger the value of R2 is, the higher the degree of interpretation of the difference by group. Pr(>F): significance. The corresponding residual term values are shown in parentheses.

Supplementary Table S5. MRPP test of community structure differences in the gut microbiota among groups after TAA injection.

| Group | A | observed-delta | expected-delta | Significance |
| --- | --- | --- | --- | --- |
| TP VS CP | 0.1916 | 0.4612 | 0.5705 | 0.001 |
| TP VS Li01 | 0.03715 | 0.4761 | 0.4944 | 0.008 |
| CP VS Li01 | 0.1385 | 0.4901 | 0.5689 | 0.001 |

MRPP: Multiple response permutation procedure; observed delta: intragroup difference; expected delta: intergroup difference. The larger the value of expected delta was, the greater the difference between the groups. A>0: intergroup difference>intragroup difference; A<0: intragroup difference>intergroup difference.
